# BCL2 Inhibition: A New Paradigm for the Treatment of AML and Beyond

**DOI:** 10.1097/HS9.0000000000000912

**Published:** 2023-06-08

**Authors:** Andrew H. Wei, Andrew W. Roberts

**Affiliations:** 1 Blood Cells and Blood Cancer Division, The Walter and Eliza Hall Institute of Medical Research, Parkville, VIC, Australia; 2 Department of Clinical Hematology, The Royal Melbourne Hospital and Peter MacCallum Cancer Centre, Melbourne, VIC, Australia; 3 Faculty of Medicine, Dentistry and Health Sciences, The University of Melbourne, Parkville, VIC, Australia; 4 Victorian Comprehensive Cancer Centre, Melbourne, VIC, Australia

## Abstract

Altering the natural history of acute myeloid leukemia (AML) in unfit and older patients has proved a highly challenging hurdle, despite several decades of concerted clinical trial effort. The arrival of venetoclax (VEN) to the clinical stage represents the most important therapeutic advance to date for older patients with AML. In this review, we will explain how and why VEN works, summarize its remarkable pathway to regulatory approval, and highlight the key milestones that have been important for its successful development in AML. We also provide perspectives on some of the challenges associated with using VEN in the clinic, emerging knowledge regarding mechanisms of treatment failure, and current clinical research directions likely to shape how this drug and others in this new class of anticancer agents are used in the future.

## VENETOCLAX: THE FIRST IN A NEW CLASS OF ANTICANCER DRUGS TO ENTER PRACTICE

**Figure 1.**
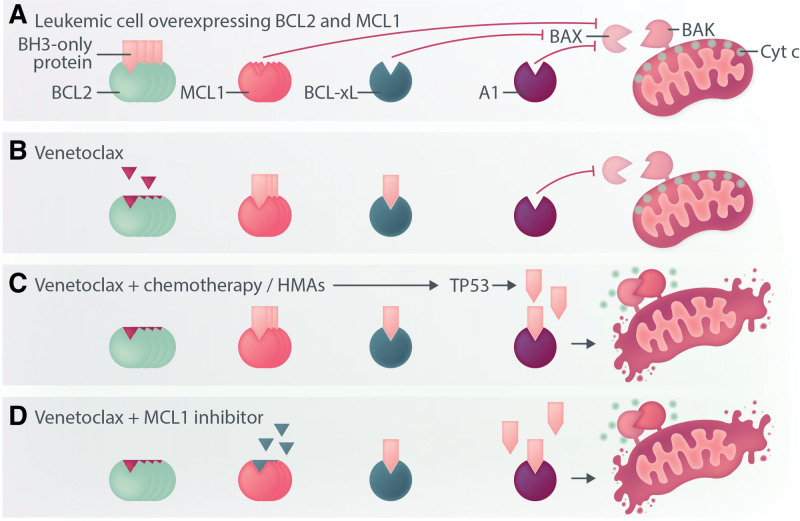
**Schematic outlining the mechanism by which venetoclax enhances the activity of cytotoxic drugs or inhibitors of MCL1.** In leukemic cells, oncogene-driven increases in BH3-only protein burden are neutralized by an increased level of BCL2 family prosurvival proteins. If prosurvival protein molecules exceed the BH3-only protein burden, BAX and BAK are kept inactive, and apoptosis inhibited (A). Venetoclax engages BCL2 binding sites, reducing the capacity for BCL2 to neutralize endogenous BH3-only proteins. Increased levels of MCL1 and other prosurvival molecules in AML may still be sufficient to prevent apoptosis (B) Chemotherapy, HMA’s (C) may further increase the total BH3-only protein load, which, in combination with venetoclax, may overwhelm the capacity of prosurvival proteins to protect the cell from apoptosis. Combining venetolax with MCL1 inhibitors (D) represents another approach to induce apoptosis in leukemic cells without requirement for DNA damage and TP53 activation. AML = acute myeloid leukemia; HMA= hypomethylating agent.

VEN is the first of a new class of anticancer drugs, so-called BCL2 homology 3 domain (BH3)-mimetics to be approved. BH3-mimetics are small molecule drugs that directly induce apoptotic cell death. Apoptosis is an evolutionarily conserved cellular switch triggered by an increase in BH3-only proteins sufficient to activate Bcl-2 Associated X-protein (BAX) and BCL2 Antagonist/Killer 1 (BAK).^1^ Once activated, BAX and BAK heterodimerize to create pores in the outer mitochondrial membrane releasing in the process, intermitochondrial membrane cytochrome *c*.^1^ The activity of proapoptotic BH3-only proteins are opposed by BCL2 family members, which include BCL2, BCL-X_L_, MCL1, and BCL2A1. A hydrophobic receptor groove on the surface of BCL2 and related proteins bind and restrain the proapoptotic function of BH3-only proteins, preventing the activation of BAX/BAK. If sufficient prosurvival BCL2 family proteins are present to neutralize ambient BH3-only protein activity, apoptosis will be avoided (Figure 1). The reason malignant cells are more sensitive to BH3-mimetic drugs than normal tissues relates to an altered equilibrium point between prosurvival and prodeath proteins shaped by constitutively determined expression of prosurvival proteins and the adaptive responses to oncogenic stresses that are intrinsically proapoptotic.^2,3^ Malignant cells, including acute myeloid leukemia (AML) blasts, often carry an increased burden of endogenous BH3-only proteins balanced by a commensurate level of prosurvival protein to neutralize the apoptotic threat posed by oncogene activation and perturbed differentiation. In these instances, the cells are primed for death and display a high level of BH3-only protein priming.^2^ In contrast, many normal adult tissues (especially brain, heart, and kidney) display a low level of BH3-only protein priming and consequently, a higher tolerance to the apoptotic effects of cytotoxic agents and BH3-mimetics.^3^ Small molecules that bind and neutralize prosurvival proteins in the same way as BH3-only proteins are called BH3-mimetics.^4^

The disclosure of VEN (ABT-199) in 2013 brought us the first BH3-mimetic that potently, selectively, and specifically inhibited BCL2 activity. It was the culmination of a 25-year international research effort that originated with the elucidation of BCL2’s prosurvival function in 1988.^5,6^ VEN was derived from the first true BH3-mimetic, ABT-737, a tool compound that inhibited all of BCL2, BCL-X_L_, and BCL-W.^7^ Targeting BCL-X_L_, however, caused acute-onset thrombocytopenia in vivo, linked to its crucial role in regulating platelet lifespan.^8^ This on-target but clinically undesirable impact on platelet viability placed limits on the potential to explore BCL2 inhibition clinically and drove the search for a selective BCL2 inhibitor.^9,10^ This culminated in the discovery of VEN, a BH3-mimetic found to specifically target BCL2 and induce BAX/BAK-dependent permeabilization of mitochondria, triggering apoptosis (reviewed in Roberts et al^11^). Although VEN had a cytotoxic effect, this new drug was unique in that it did not cause DNA damage, nor interfere with mitogenesis.

Preclinical studies dating back to 2006 had already indicated that ABT-737 had proapoptotic activity in AML.^12^ Furthermore, the activity of ABT-737 was greatly enhanced by concurrent targeting of MCL1, signaling potential for broad application among hematologic malignancies.^13^ In 2012, Glaser et al^14^ employed elegant gene targeting systems to reveal the importance of Mcl1 in mediating AML survival, whereas Vo et al^15^ demonstrated that ABT-737 could induce apoptosis more effectively in leukemic, compared with normal CD34+ progenitors. Cotargeting BCL2 with ABT-199 and MCL1 via expression of an engineered BH3-mimetic ligand proved synergistic in xenograft models of AML and indicated that robust activity in AML would likely require combination therapy.^16^ Multiple laboratories demonstrated that combination of ABT-199 with anthracyclines, cytarabine or hypomethylating agents (HMAs), were all potentially effective options.^15–17^ The clinical investigation of BCL2 in AML, however, was only feasible after the safety and efficacy of VEN was first established in patients with chronic lymphocytic leukemia.^18^

## INITIAL OBSERVATIONS OF VEN AS A SINGLE AGENT IN AML

The initial phase 2 study administered VEN 800 mg daily to 32 patients with predominantly relapsed/refractory AML.^19^ This heavily pretreated population included 53% who had failed prior 7 + 3 induction, 75% had prior exposure to HMAs, and 41% had received 3 or more prior lines of therapy. After the first month of therapy, over one-third had a ≥50% reduction in marrow blasts, with International Working Group complete remission/incomplete remission (CR/CRi) responses achieved in 19% (CR 6%) associated with a median response duration lasting <6 months. No episodes of tumor lysis syndrome (TLS) were observed.

Despite the escalation of VEN to 1200 mg in 14 of 32 cases failing to respond to the initial dose of 800 mg, additional antileukemic activity was not observed, suggesting again that BCL2 inhibition alone would be insufficient for treatment of AML. Of 12 patients in the study with *IDH* mutation (10 involved IDH2), 4 of 12 (33%) responded to VEN. Interestingly, among patients with paired bone marrow samples available, 7 of 10 with an *IDH1/2* mutation showed an interval reduction in bone marrow blasts. In contrast, responses were not observed among those with either *FLT3*-ITD or *PTPN11* mutant AML at baseline. In addition, 4 patients lacking detectable *FLT3*-ITD at study entry had *FLT3*-ITD emerge by the end of therapy, suggesting the potential for activating kinase variants to provoke VEN resistance.^20^ Although the number of cases in this study were small, the general patterns identified in these early studies have proved valid.

## LESSONS LEARNED FROM COMBINING VEN WITH LOW-DOSE CYTARABINE OR HYPOMETHYLATING AGENTS

Despite only modest efficacy being observed in the phase 2 study with VEN, further exploration continued in combination with either HMAs or low-dose cytarabine (LDC), this time in the frontline setting, among older patients considered unfit for intensive chemotherapy. Although preclinical data existed to support the rationale for these combinations, the basis for these clinical studies were largely empirical and based on adding VEN to existing standards of care.^16,21^

Upon activation of these studies around 2014, the clinical bar for elderly AML was low, with azacitidine (AZA) or LDC associated with response rates between 19% and 28% and median survival expectation only 4–10 months.^22,23^ As a result, clinical studies involving VEN began at a time when the prevailing clinical attitude was that of therapeutic nihilism, with many patients >65 years managed with palliative intent from the outset.^24^

The first results for VEN in combination with hypomethylating agents (AZA or decitabine) as first-line therapy including 57 patients with a median age of 75 years and only 12 months follow-up exceeded clinical expectations.^25,26^ TLS risk mitigation measures employed in the protocol included baseline white cell count cytoreduction to <25 × 10^9^/L, prehydration, prophylactic uricosuric agents, a 3–4 day VEN dose ramp-up and close postdose biochemical monitoring. Based on this practice, episodes of biochemical TLS, a problem encountered in the early days of chronic lymphocytic leukemia (CLL) treatment with VEN,^18^ were extremely rare. After a 3-day dose ramp-up, VEN 400 mg was administered daily until the first marrow assessment on day 28. Bone marrow blast clearance (to <5%) was achieved in >70% of cases, with CR/CRi responses recorded in 60%. Notably, blast clearance was rapid and achieved after a median of just 1 cycle, much faster than historically expected for HMAs or LDC.^25^

The combination of VEN and HMA was myelosuppressive, with the next cycle of therapy frequently delayed until resolution of grade 4 neutropenia and thrombocytopenia. A frequent observation after documentation of bone marrow blast clearance was the surprisingly prompt resolution of severe neutropenia if VEN dosing was interrupted and granulocyte colony stimulating factor (G-CSF) commenced. Initial indications were that hematologic responses were durable (median, 11.0 months) and associated with encouraging survival outcomes (median, 15.2 months). Subsequent analysis of an expanded cohort of patients (n = 145) with 15 months of median follow-up saw these early observations consolidated.^26^

A parallel study combined LDC with VEN 600 mg in 82 elderly patients with AML. One major point of difference in the eligibility criteria was inclusion of patients with prior HMA exposure (29%) who were excluded from the parallel HMA + VEN study. Among patients without prior HMA exposure, the CR/CRi rate achieved with LDC + VEN was 62%, with median response duration 14.8 months and overall survival (OS) 13.5 months.^27^ Outcomes for patients with prior HMA exposure were poor, with only 33% responding to treatment and median OS only 4.1 months. Based on the promising results from these 2 open-label studies and the high unmet need at the time for older patients with AML, the US Food and Drug Administration granted accelerated approval on November 21, 2018 to VEN, in combination with either AZA, decitabine, or LDC, for treatment of newly diagnosed AML in adults aged ≥75 years or younger patients in the presence of comorbidities precluding use of intensive induction chemotherapy.

Based on the prediction that CYP3A4 inhibitors would lead to increased levels of VEN, a pharmacokinetic study was performed in 12 patients whereby posaconazole was coadministered with VEN on days 21–28 of the cycle. Compared with VEN 400 mg, coadministration of posaconazole with VEN 50 or 100 mg resulted in a 53% or 93% increase in C_max_ and a 76% or 155% increase in area under the curve (AUC)_0-24_, respectively, by day 28.^28^ Overall, VEN was estimated to increase VEN C_max_ and AUC_0-24_ by 7.1-fold and 8.7-fold, respectively. In other words, in the presence of posaconazole, VEN 50 or 100 mg would be equivalent to a dose of 400 or 800 mg in the absence of a strong CYP3A4 inhibitor, respectively.^28^ In the dose finding phase of the HMA+ VEN phase 1b of 2 study, although VEN 800 and 1200 mg dose levels were tolerable, in comparison to 400 mg per day, longer times to blood count recovery were observed in subsequent cycles.^25^ A similar observation was made for LDC + VEN at the 800 mg dose level, compared with 600 mg per day.^27^ Therefore, in both the HMA and LDC + VEN studies, posaconazole was administered with an adjusted VEN dose of 50 mg per day.

## A POSITIVE PHASE 3 OUTCOME AND THE IMPACT ON CLINICAL PRACTICE

Based on the encouraging phase 1b of 2 study results, 2 parallel phase 3 studies were designed to test the addition of VEN to standard regimens for older or unfit patients with AML, with OS as their primary end point.

The VIALE-C study enrolled 211 patients in a 2:1 ratio to either LDC or placebo in combination with VEN. At the time of the preplanned primary analysis and with only 12 months of median follow-up time, OS in the LDC plus VEN arm was not significantly greater than LDC plus placebo (hazard ratio [HR], 0.75; *P* = 0.11), despite a significantly enhanced response rate (CR/CRi, 48% versus 13%). With 6-month additional follow-up, a post hoc analysis revealed OS was in fact greater in the VEN arm (median 8.4 versus 4.1 months; HR 0.70 [95% confidence interval [CI], 0.50-0.98; *P* = 0.04]).^29^ A final follow-up performed with 34.7 months of median follow-up time demonstrated 2-year OS to be 21.5% in the LDC+ VEN arm, compared with 12.4% for patients in the LDC + placebo arm (number needed to improve survival at 2 years of 11). The best performing subgroup receiving LDC+ VEN were patients with an *NPM1* mutation, who had a response rate of 78% and median OS exceeding 2 years.^30^

The VIALE-A study enrolled 431 patients, also in a 2:1 ratio, to either AZA or placebo in combination with VEN. With a median follow-up time of 20.5 months, the primary analysis of the VIALE-A study was positive, with median OS in the AZA plus VEN arm 14.7 months, compared to 9.6 months in the placebo arm (HR, 0.66 [95% CI, 0.52-0.85]; *P* < 0.001), with a substantially improved response rate (CR/CRi, 66% versus 28%).^31^ A long-term follow-up analysis with 43.2 months of median follow-up time showed continued separation of the survival curves, with 3-year OS ~38% in the AZA+ VEN arm, compared with 20% for patients in the AZA + placebo arm (number needed to improve survival at 3 years of ~5.6).^32^ Analogous to the phase 1b/2 experience, CR/CRi responses after VEN-AZA were achieved rapidly, with 65%, 75%, and 93% of responses achieved after 1, 2, and 4 cycles of therapy.^33^

## THE ART OF USING VEN-AZA IN THE CLINIC

As discussed earlier, VEN is the first of a new class of anticancer drug and BCL2, a new target. Consequently, we should not expect that the patterns of responses and toxicities familiar to clinicians in association with DNA-damaging or HMAs will necessarily apply when using VEN-based combinations. Not surprisingly, the transition of VEN-AZA from the strictly regulated environment of clinical trials into routine clinical practice has been more challenging than any other recently approved drug in AML. Indeed, the limitations associated with clinical trials could not have prepared us fully for decisions around how best to use this drug for treatment of AML in day-to-day practice.

Concern regarding the risk of TLS in the early days of clinical development led to implementation of highly conservative treatment practices. In clinical trials, the frequency of TLS has been very low (1%–6%).^31,34^ Based on this low risk, there has been strong incentive to commence VEN-based therapy in the outpatient setting. In the context of a single-institution real-world study, 5.4% patients receiving VEN-based therapy had evidence of biochemical TLS with laboratory values outside the institutional reference range, with 2.7% meeting criteria for clinical TLS.^35^ A multivariate analysis identified presence of *IDH2* mutation and elevated baseline lactate dehydrogenase as risk factors for TLS. In our experience, some patients may have evidence of TLS in the absence of any clear risk factor, such as increased lactate dehydrogenase, high bone marrow burden, or elevated circulating white cell count (WCC). A particularly dangerous acute manifestation of TLS in AML is an acutely elevated serum potassium level that may occur as early as 4–6 hours after the first ramp-up dose of VEN. For each patient, therefore, it is prudent to make sure the WCC before treatment commencement is reduced to <25 × 10^9^/L, elevated potassium levels prophylactically ameliorated, and uricosuric agents commenced. Although TLS is rare, it is imperative that each patient have a biochemical check to assess for TLS complications ~6 hours after each ramp-up dose and to rule out extreme hyperkalemia before discharge from care. In regard to the need for hospitalization, patients with a high risk of TLS should be considered for admission until completion of the ramp-up phase. For patients with inadequate social supports, burdensome comorbidities, or excessive frailty, inpatient management until first remission and associated marrow recovery should be strongly considered.

Dosing adjustments are commonly required in clinical practice and several points are worth highlighting. First, noting the pharmacokinetic data, and if a strong CYP3A4 inhibitor (eg, posaconazole) is used, our preference is to reduce the VEN dose~ 8-fold (eg, to 50 mg/d if the unadjusted VEN dose was 400 mg). Second, analysis of the VIALE-A study showed that 60% of patients achieving CR/CRh had their duration of VEN dosing reduced from 28 to 21 days (approximately half switching in the cycle following response and the remainder after a median of ~3 cycles). Only 10% patients maintained a 28-day VEN schedule. OS outcomes were comparable between patients moving to a 21 of 28-day schedule early versus later.^36^ As a result, our practice is to perform a bone marrow around day 21 and, if marrow blast clearance has been achieved, to cease VEN and commence G-CSF if the absolute neutrophil count is <0.5 × 10^9^/L. Use of postremission G-CSF in the VIALE-A study appeared to shorten the duration of postremission grade ≥3 neutropenia from 16 to 12.5 days, without compromising OS outcome, compared with patients not given postremission growth factors (OS 83% versus 71% at 12 mo).^37^ The next cycle of therapy is preferably not started until the neutrophil count recovers to grade 2 (≥1 × 10^9^/L) and the platelet count to grade 1 (≥75 × 10^9^/L) severity; this may require up to 2 weeks to achieve.

Third, in contrast to HMAs alone, VEN works very rapidly in AML. For patients with *NPM1* or *IDH2* mutant AML, bone marrow blast reductions >50% in magnitude may be observed after just 7 days exposure to single-agent VEN.^38^ For patients receiving VEN-AZA, 76% of those destined to achieve blast clearance will do so after the first cycle of therapy. If no reduction in marrow blasts is evident after 2 cycles of therapy, ongoing cycles of treatment are unlikely to deliver significant benefit but very likely will cause toxicity and, hence, we would consider clinical trials or alternative treatment options at this point. If treatment has produced an interval reduction in marrow blasts, or an improvement in blood counts, continuation of therapy is justified, as another 15%–20% will achieve response by the end of cycles 3–4. Furthermore, survival outcome for patients with an early objective response (CR/CRi within 56 days) appears similar to patients achieving a later response.^33^

Fourth, the most challenging management scenario entails patients with prolonged grade 4 neutrophil and/or platelet toxicity, despite bone marrow blast clearance. A feature of both VIALE-A and VIALE-C studies was the higher rate of grade 3 or higher neutropenia in the VEN arm compared with the control group (VIALE-A 42% versus 28% and VIALE-C 46% versus 16%), as well as grade 3 or higher febrile neutropenia (VIALE-A 42% versus 19% and VIALE-C 32% versus 29%), confirming the myelosuppressive nature of these new combination regimens. In our experience, occurrence of severe gastrointenstinal mucosal toxicity after VEN-based induction was notably low, compared with prior experience with intensive chemotherapy. After achieving remission, if recovery from treatment-related grade 4 neutropenia or thrombocytopenia is quite delayed, subsequent cycles may be delivered with shorter duration exposures of VEN, for example, from 21 to 14 days. The actual dose of VEN is generally not reduced unless related to a drug-drug interaction. Prolonged cytopenia is more likely to occur after several cycles of therapy in patients with preexisting myelodysplastic syndrome or extensive myelofibrosis. For such patients, consideration should be given to commencing VEN-AZA using an abbreviated 14-day treatment schedule, along with prophylactic medications to mitigate the risk of septic and fungal complications.^39^ For patients with prolonged cytopenias related to severe bone marrow hypocellularity, consideration should be given to reducing the dose of AZA or even deferring further therapy altogether.

## MECHANISMS OF TREATMENT FAILURE

Despite the high response rates observed with VEN-based therapy, long-term follow-up studies suggest that most patients will ultimately relapse, with projected 4-year survival currently estimated to be ~15% after VEN-AZA.^32^ The activity of VEN is dependent on its ability to bind 2 hydrophobic residues in the BH3 binding groove (P4 and P2).^5^ Confirming the relevance of these residues to VEN activity, clinical resistance in patients with CLL treated with VEN has been linked to emergence of on-target *BCL2* mutations disrupting the fidelity of VEN engagement to both the P4 (D103T/G/V) and P2 (V156D) binding pockets.^40^ Although *BCL2* mutations linked to treatment resistance have been reported in up to 50% of patients with CLL progressing while on long-term (ie, >2 y) VEN, such mutations appear rare in AML.^41^ This may relate to the rapidly progressive nature of AML, such that fewer patients are exposed to long-term VEN monotherapy, and the different cell contexts that influence mutational activity.

**Figure 2.**
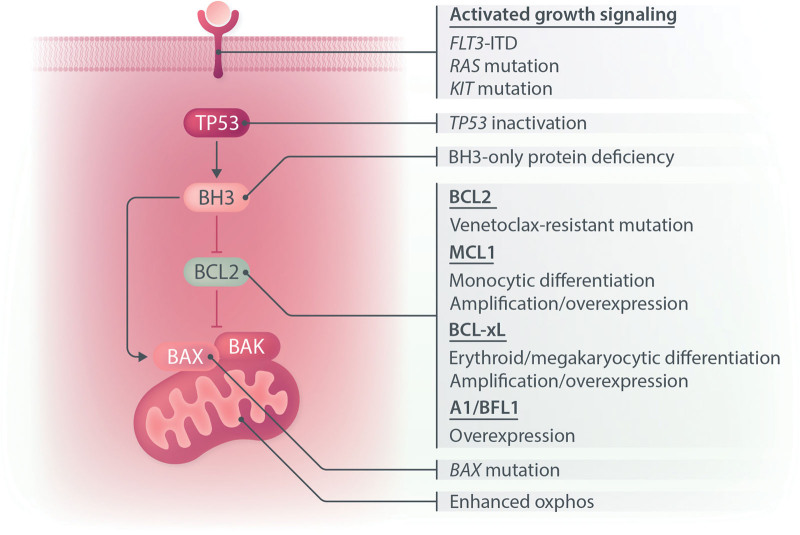
**Selected mechanisms of venetoclax resistance reported in AML.** AML = acute myeloid leukemia.

In patients with AML relapsing after VEN-based therapy in combination with HMA, LDC, or intensive chemotherapy, resistance mechanisms affecting VEN activity have been identified at multiple levels of the intrinsic apoptosis cascade (Figure 2). This includes somatic activation of the receptor tyrosine kinase pathway (eg, by *FLT3*-ITD, *N/KRAS*, or *KIT* mutation), which enhance proliferation by replicating growth factor stimulation,^42,43^ multi-hit inactivation of *TP53*,^42,44^ BH3-only protein deficiency,^45^ selection of monocytic lineage blasts with elevated levels of endogenously expressed MCL1,^46^ erythroid/megakaryocytic differentiation conferring dependency on prosurvival BCL-X_L_,^47^ amplified expression of MCL1,^48^ BCL-X_L_, or BCL2A1,^48,49^ or inactivating mutations affecting BAX expression or function.^41,50^

Many processes associated with VEN resistance converge on enhanced expression of prosurvival partners not directly targeted by the BCL2 inhibitor. Activating *FLT3* and *RAS* mutations, for example, may result in enhanced MCL1 expression and sensitivity to MCL1 targeted drugs.^51,52^ Small molecule inhibitors of oncogenic kinases, for example FLT3, may suppress MCL1 expression and combine synergistically with VEN to enhance antileukemic activity.^42^ As discussed later, several studies are showing early promise from the combination of FLT3 inhibitors with VEN in patients with *FLT3*-mutated disease.

In the setting of *TP53*-mutated AML, clinical experience suggests that outcomes are not substantially improved by VEN in combination with either chemotherapy or HMAs.^38,53,54^ Although higher initial responses may be achieved with this therapy, clinical relapse and treatment failure is inevitable in *TP53*-mutated AML. Longitudinal studies show strong clonal selection for multi-hit *TP53* defects, in the form of mutant *TP53* hemizygosity or expansion/acquisition of multiple *TP53* variants.^42^ Despite an elevated threshold for activating apoptosis in *TP53* defective AML,^44^ sensitivity to BH3-mimetics may be restored by concomitant small molecule targeting of BCL2 and MCL1, which appear highly synergistic in combination and effective at suppressing TP53 defective AML in vitro and in vivo.^44,55,56^ Early clinical experience with MCL1 inhibitors, however, has been hampered by biochemical elevations in serum troponin, with clinical investigation ongoing to determine the feasibility of combining MCL1 inhibitors with VEN and HMAs.

Proapoptotic BAX is normally located in the cytosol, until activated by BH3-only proteins, resulting in translocation of the molecule to the mitochondrion and insertion of the alpha 9 hydrophobic tail of BAX into the mitochondrial outer membrane (MOM). Expansion of leukemic clones harboring mutant *BAX* have been observed in patients receiving VEN for AML and similar variants have emerged in the pre-leukemic myeloid compartment of patients receiving VEN for CLL.^57,58^ These VEN-resistant *BAX* variants may either take the form of indels resulting in a nonexpressed or truncated protein, or missense lesions impairing the ability of BAX to insert into the MOM. Functionally, defective BAX causes resistance of cells to BH3-mimetics. Therefore, in the presence of BAX loss of function defects, it is predicted that BH3-mimetic combinations alone are unlikely to be efficacious.

In addition to inducing apoptosis, VEN has been reported to perturb the function of leukemic stem cells (LSCs) by interfering with oxidative metabolism.^59–61^ Resistance to VEN has been reported in association with processes augmenting the supply of metabolic substrates driving cellular respiration, such as the increased uptake of amino or fatty acids. Cross-talk may exist between changes in cellular metabolism and gene mutations associated with VEN resistance. For example, mutated *RAS* has been linked to enhanced fatty acid oxidation and deficient TP53 has been linked to enhanced electron transport accompanied by an increase in cellular reactive oxygen species.^45,62^ Decreases in oxygen consumption normally induced by VEN, however, are not observed in BAX/BAK deficient cells, suggesting that changes in cellular metabolism may rely, in part, on mitochondrial permeabilization as a consequence of BAX/BAK activation.^63^

## PROGNOSTIC VERSUS PREDICTIVE BIOMARKERS FOR VEN USE IN AML

A recent analysis of the VIALE-A study found that both the ELN 2017^64^ and ELN 2022^65^ risk classifications failed to clearly stratify survival for patients receiving VEN-AZA, indicating that the ELN risk model, which was developed in younger patients receiving intensive chemotherapy, was not fit for purpose when applied to older patients with AML receiving VEN-AZA.^66^ A sequential-BATTing (bootstrapping and aggregating of thresholds from trees) approach was used to stratify patients receiving VEN-AZA into 3 prognostic risk groups, defined by the presence or absence of *TP53*, *FLT3*-ITD, and K/NRAS mutations (Table 1).^66,67^ Consistent with prior publications, prognosis was worst for patients with mutated *TP53*, who were designated as a lower benefit subgroup, with median OS <6 months.^42^ Patients who were *TP53* wild-type, but *FLT3*-ITD or K/NRAS mutated had a median OS of ~1 year and were designated to have intermediate benefit. Finally, patients lacking either *TP53*, *FLT3*-ITD, or K/NRAS variants were found to have a median OS of over 2 years and designated as a higher benefit group. Interestingly, within this higher benefit group, patients with *NPM1* mutation had a median OS expectation exceeding 3 years (39 mo), compared with other patients with higher benefit where median OS ranged from 23 to 33 months.^42^ Comparing the survival outcome between VEN-AZA and AZA within each of the 3 risk categories demonstrated improved survival for patients receiving VEN-AZA in the higher benefit group, suggesting it to be a predictive biomarker for VEN-AZA therapy (Table 1). In contrast, outcomes for patients in the other risk categories did not appear significantly improved by VEN.

**Table 1:** Molecular Factors Impacting Outcome Among Patients Receiving Venetoclax-azacitidine

Biomarker Profile^66^	Prognostic	Median OS (95% CI) for VEN-AZA	Predictive	HR for OS relative to AZA (95% CI)
Higher benefit TP53^WT^, No FLT3-ITD, K/NRAS^WT^	Yes	26.51 mo (20.24-32.69)	Yes	HR 0.37 (0.27-0.52)
Intermediate benefit TP53^WT^ and FLT3-ITD or K/NRAS mutated	Yes	12.12 mo (7.26-15.15)	No	HR 0.71 (0.44-1.16)
Lower benefit TP53 mutated	Yes	5.52 mo (2.79-7.59)	No	HR 0.72 (0.42-1.24)

AZA = azacitidine; CI = confidence interval; HR = hazard ratio; OS = overall survival; VEN = venetoclax.

The prognostic separation of older patients with AML into 3 prognostic risk strata raises important issues for clinical practice. For patients with higher benefit, with expected median survival extending beyond 2 years, and in the case of *NPM1* mutation beyond 3 years, an important question is whether recurrent cycles of VEN-AZA therapy should be delivered until progression, or whether a similar prognosis could be achieved using time-limited therapy, with quality of life enhanced via a period of treatment-free remission. A small exploratory study (n = 29) compared patients who had received at least 12 months of therapy and treatment either ceased in remission or continued until progression. Patients who ceased therapy had a median treatment-free remission of 45.8 months, with the risk of relapse and OS similar between the stop and continuation groups.^68^ In another study (n = 51), where half the patients had VEN +/− AZA ceased, the median treatment-free remission observed was 10 months. Interestingly, for 12 patients who resumed VEN and/or AZA treatment, the rate of second remission was 50%.^69^ In both the studies, the importance of evaluating MRD before ceasing therapy was emphasized.

Based on the clinical concern expressed by physicians regarding myelosuppression associated with VEN-AZA, several single-arm studies have explored truncated VEN schedules, including a 7-day VEN-AZA treatment regimen.^70^ An even more extreme metronomic schedule administering only a single dose of decitabine and VEN weekly has also been proposed.^71^ Although early response rates appear comparable, it remains unclear if these shortened regimens will match long-term outcomes observed in VIALE-A, especially for those in the higher-benefit group, where the clinical benefit of adding VEN is highest.

## EMERGING DIRECTIONS FOR BCL2 TARGETED THERAPIES IN AML

**Figure 3.**
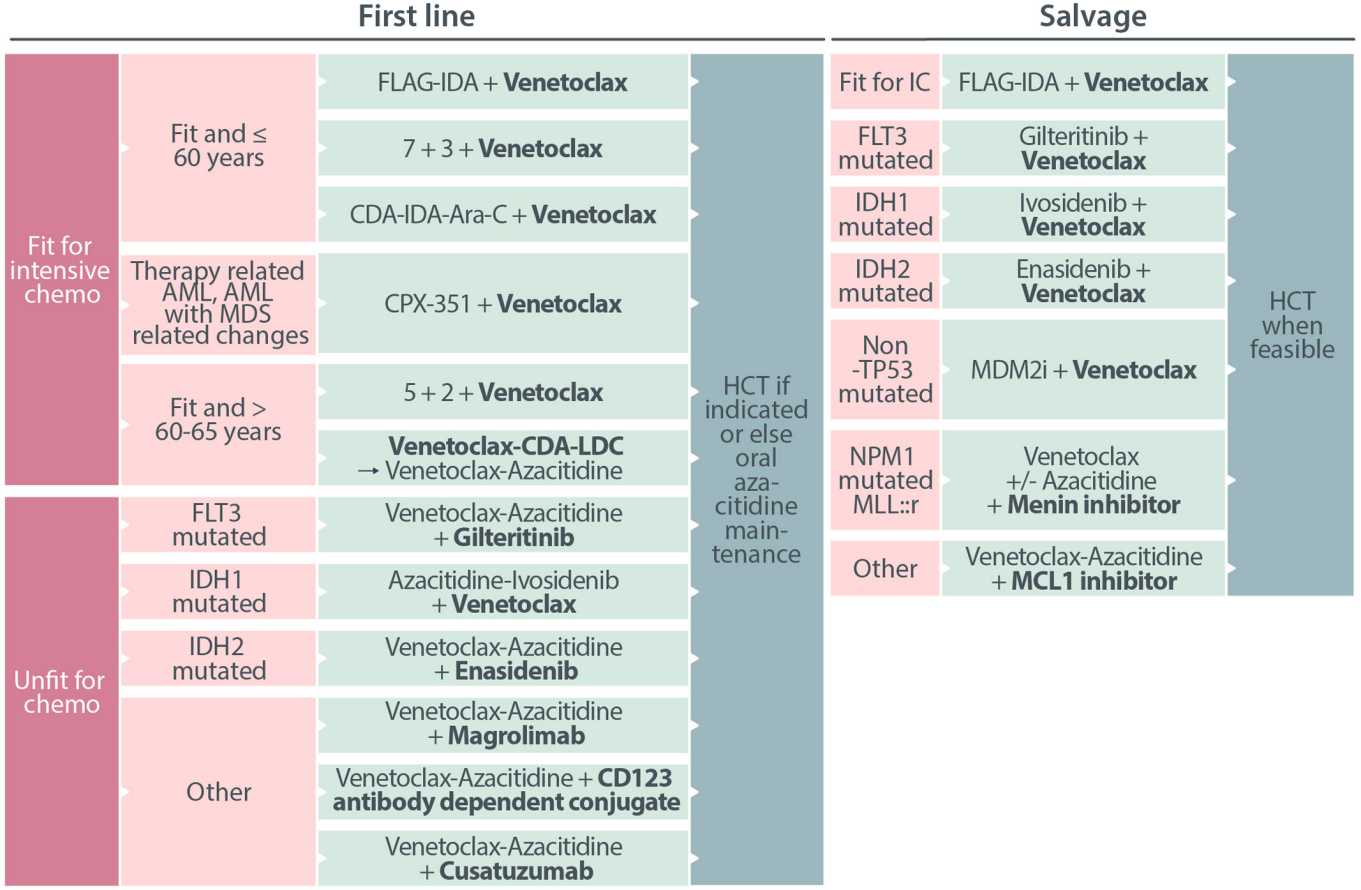
**Selected venetoclax-based combination studies in clinical development.** ADC = antibody dependent conjugate; AML = acute myeloid leukemia; AML-MRC = AML with MDS related changes; AZA = azacitidine; IC = intensive chemotherapy; MCL1i = MCL1 inhibitor; mut = mutated; t-AML = therapy-related AML; VEN = venetoclax; y = years.

After the approval of VEN for AML, the number of clinical studies aiming to incorporate VEN into conventional or novel drug regimens for use in AML has increased rapidly (Figure 3). Several general approaches have been explored. These include the following: (1) combining VEN with intensive chemotherapy; or (2) combining VEN with novel agents either as doublet or triplet combinations with VEN-AZA.

### VEN and intensive chemotherapy

The tolerability of VEN (50–600 mg) in combination with intensive chemotherapy (5 + 2) was initially investigated in patients aged 65 years and over in a dose finding study (CAVEAT).

The main observations from this study were as follows: (1) induction with 5 + 2 + VEN in fit older patients was efficacious (CR/CRi, 72%); (2) the median time to neutrophil (≥0.5 × 10^9^/L) and platelet recovery (≥50 × 10^9^/L) were in the range expected for chemotherapy alone (26 days); (3) a dose-response relationship was observed for CR, reaching a plateau with VEN doses ranging between 200 and 600 mg; and (4) outcomes were best among patients with *IDH2* and *NPM1* mutant AML and worst among those with *TP53* mutation.

The MD Anderson group have incorporated VEN into several multiagent treatment regimens with the goal of demonstrating feasibility and improving response via the leveraged addition of VEN (Table 2). For patients >60 years, VEN was added to cladribine/LDC and this triplet regimen alternated with VEN-AZA.^73^ For patients predominantly <60 years, VEN has also been added to either fludarabine, high-dose cytarabine, G-CSF and idarubicin^74^ or cladribine, idarubicin, and high-dose cytarabine.^77^ For all these regimens, CR/CRi response rates exceeded 90% in the first-line setting, with most patients experiencing neutrophil and platelet recovery within 6 weeks. After the second cycle of therapy, neutrophil and platelet recovery were longer, with full platelet recovery a greater issue than neutrophil recovery. These augmented regimens enabled a high proportion of patients to proceed to allogeneic stem cell transplantation (SCT), while also achieving excellent disease control, as measured by flow MRD clearance. Collectively, these studies confirm the feasibility of combining VEN with intensified chemotherapy, but also highlight the cumulative risk from delivering repeated cycles of VEN plus chemotherapy in consolidation, suggesting these strategies are best suited to patients for whom allogeneic SCT will be the definitive therapy.

**Table 2:** Selection of Studies Examining the Efficacy of Venetoclax in Combination With Chemotherapy

Regimen	Setting	Median Age, y (Range)	N	CR + CRi	Median OS	Allo-HCT	Median Follow-up
Median age >60 y							
** **5 + 2 plus VEN^38,72^	First line	72 (63–80)	69	73%	15.4 mo	1%	37 mo
** **Cladribine-LDAC VEN-AZA^73^	First line	68 (57–84)	60	93%	Not reached	48%	22 mo
**Median age ≤60** y							
** **FLAG-Ida-VEN^74^	First-line Salvage	46 (20–63) 47 (22–66)	29 23	90% 61%	Not reached Not reached	69% 46%	12 mo
** **CLIA + VEN^75^	First line	48 (18–64)	50	94%	Not reached	32%	14 mo
** **7 + 3 + VEN^76^	First line	40 (18–60)	33	91%	Not reached	36%	11 mo

AZA = azacitidine; CLIA = cladribine, idarubicin and high-dose cytarabine; FLAG-Ida = fludarabine, high-dose cytarabine, G-CSF and idarubicin; OS= overall survival; VEN = venetoclax.

### VEN combined with novel agents

Prosurvival dependency in AML is highly heterogeneous. Cell differentiation state may influence BCL2 family expression, such as enhanced MCL1 expression in monocytic cells,^46^ or BCL-X_L_ expression in cells of erythroid and megakaryocytic lineage.^47^ Leukemic mutations may tilt dependence toward certain prosurvival members, such as BCL2 in the presence of mutated *NPM1* or *IDH*, or MCL1 in the presence of oncoactivated *FLT3* or *RAS* kinases.^38,42,43^ Analogous to the multiple ancestral and leukemic subclones that contribute to the multiclonal diversity of AML, single-cell technologies have revealed the leukemic milieu to be composed of a multitude of subpopulations manifesting varied expression and function of individual proapoptotic and prosurvival components. Furthermore, upon application of selective pressure, rapid and dynamic evolution of resistant subpopulations may occur, leading to drug resistance and treatment failure. Therefore, although we can expect enhanced activity if effective targeted drugs are combined, it should also be expected that polyclonal evolution of targeted drug resistance will be the norm (Figure 3 and Table 3).^86^

**Table 3:** Selection of Clinical Results Examining Venetoclax in Combination With Other Novel Drugs

Regimen	Setting	Median Age, y (Range)	N	CR + CRi CRp	Median OS	Median Follow-up
Gilteritinib + VEN^78^	*FLT3* mut- salvage	63 (21–85)	61	40%	10 mo	17.5 mo
VEN-AZA + Gilteritinib^79^	*FLT3* mut- first-line older/unfit Salvage	70 (18–86) 69 (19–90)	27 20	96% 35%	Not reached 5.8 mo	12 mo
Ivosidenib + VEN^80^	*IDH1* mut- salvage First-line older/unfit	67(44–84)	6 4	67% 100%	9 mo 8 mo	24 mo
Ivosidenib + VEN + AZA^80^	*IDH1* mut- salvage First-line older/unfit	N/A	2 10	50% 90%	7.5 mo Not reached	24 mo
Enasidenib + VEN^81^	*IDH2* mut- salvage	72 (32–80)	11	45%	Not reached	N/A
VEN-AZA + Enasidenib^82^	*IDH2* mut- salvage First-line older/unfit	64 (24–88) 77 (66–81)	19 7	58% 100%	Not reached Not reached	11.2 mo 13.1 mo
VEN-AZA + Magrolimab^83^	*TP53* mut first-line older/unfit	65 (33–84)	27	63%	10.4 mo	14 mo
VEN-LDC + midostaurin^84^	Non-adverse karyotype, first-line older/unfit	77 (73–87)	18	78%	Not reached	18 mo
VEN-AZA + Cusatuzumab	First-line, older/unfit	75 (32–89)	44	77%	Not reached	7 mo
Idasanutlin-VEN^85^	Salvage	72 (62–93)	50	26%	5.1 mo	3.9 mo

AZA = azacitidine; N/A= not available; VEN = venetoclax.

The concept of combining BH3-mimetics with drugs targeting activated mutant kinases is strongly supported preclinically, as exemplified by ABT-737 in combination with imatinib in *bcr-abl* mutant cells,^87^ VEN in combination with ibrutinib in mantle cell lymphoma,^88^ and VEN combined with FLT3 inhibitors in *FLT3* mutated AML.^42,89^ Clinically, VEN-gilteritinib is the most advanced BCL2-kinase inhibitor combination in development for AML.^78^ Key observations from this study include the general tolerability of the combination, apart from myelosuppression, which resulted in most responses being morphologic, rather than complete. Among 61 patients who received VEN-gilteritinib in a phase 2 study, grade 3 or 4 cytopenia was experienced by 80%, with ~50% experiencing febrile neutropenia and the same proportion requiring VEN-gilteritinib dose interruptions. Although CR/CRi/CRp responses were observed in 40%, another 36% had morphologic leukemia-free state (MLFS) as best response. The long half-life of gilteritinib is a potential contributor to delayed marrow recovery when combined with VEN. Despite the higher rate of overall response, the median OS of 10 months in relapsed/refractory FLT3 mutated AML did not appear, on face value, to represent a marked improvement over the median OS reported for gilteritinib as a single agent in the ADMIRAL study.^90^

Based on the evidence that neither VEN nor gilteritinib, when combined with AZA, appear to improve OS in *FLT3*-ITD AML, the combination of VEN-AZA-gilteritinib as a frontline triplet regimen has been explored.^79^ Preliminary findings to date among patients with *FLT3*-ITD (n = 19) indicate a high response rate, with estimated 12-month OS 79%. The major limitation has been delayed neutrophil and platelet recovery, resulting in truncation of the VEN duration to 7 days, the AZA to 5 days, and a reduction in the dose of gilteritinib from 120 to 80 mg per day. It remains to be determined whether frontline VEN-AZA-gilteritinib will alter the natural history of *FLT3*-ITD AML despite the failure of prior AZA doublets for this indication.

VEN+AZA is also being explored as the backbone for an expanding array of triplet combinations with small molecules targeting IDH1,^80^ IDH2, MDM2,^85^ or menin (NCT05453903); antibodies targeting CD47,^83^ CD70,^91^ TIM3 (NCT04150029) or CD123;^92^ and other BH3-mimetics targeting MCL1 (NCT03672695, NCT03797261, and NCT03218683) (Figure 3). Despite the growing flurry of clinical activity associated with the development of VEN-AZA triplets, substantial challenges exist with this approach. For *FLT3*, *IDH1*, or *IDH2* mutated AML, in addition to the increased difficulty of recruiting sufficient patients to a targeted subgroup, the availability of the same targeted drugs in salvage could compromise the likelihood of improving OS. If event-free survival is instead used as a primary end point, achievement of CR is often a critical determinant of success. If multiagent VEN-based regimens exacerbate the occurrence of prolonged cytopenias, documentation of true CR will be reduced and this may artificially reduce apparent effectiveness as failure to achieve a true CR would be considered an event.

## DEVELOPMENT OF NOVEL BH3-MIMETICS TARGETING ALTERNATIVE PROSURVIVAL PROTEINS

An important theoretical advantage of the BH3-mimetic class of drugs over DNA-damaging chemotherapy is their mechanism of action downstream of TP53; that is, they can kill TP53-aberrant malignant cells. Although defective TP53 function may raise the apoptotic threshold for activation by a single BH3-mimetic, combined targeting of BCL2 and MCL1 appears to circumvent this issue and is currently being explored in several phase 1 studies.^44^ This approach has strong appeal for patients with mutated *TP53*, as effective treatment options are lacking for this poor risk subgroup.

Although several MCL1 inhibitors have entered clinical development (S64315 [Servier], AMG-176 and AMG-397 [Amgen], AZD5991 [Astra Zeneca] and PRT1419 [Prelude]), cardiac safety remains a primary concern, as biochemical increases in troponin have been observed as a likely class effect, highlighting the known potential for on-target cardiomyocyte toxicity previously characterized in Mcl1 knock out mice.^93,94^ Heterozygous *mcl-1* (+/−) mice, however, appear healthy and unaffected, suggesting a therapeutic window may be feasible, especially if the dose of MCL1 inhibitors can be kept below the threshold for toxicity to nonmalignant tissues.^14^ This will require combination of MCL1 inhibitors with other drugs lacking an overlapping cardiotoxicity signal.

## IMPACT ON PRACTICE AND FUTURE QUESTIONS

In the space of just over 5 years, the therapeutic landscape of AML has witnessed dramatic change. Over half the AML population is aged 65 years or over at diagnosis. Clinical progress for patients unfit for intensive chemotherapy seemed almost unachievable barely a decade ago. Before introduction of VEN, for patients aged >65 years with AML, no active antileukemic therapy was offered to ~1 of 3 patients in the US and Europe, with the OS expectations ranging between 1.2 and 4.8 months.^95^ Over the last 15 years, the genomic structure of AML by next-gen sequencing made the potential for parallel advances in AML therapy seem highly challenging, especially for the patient population considered unfit for intensive chemotherapy.^96,97^ The elevation of VEN to its current role in AML has required a series of major barriers to be hurdled. These include the development of novel compounds to disrupt complex protein-protein interactions,^7^ further chemical modifications to identify a BH3-mimetic with selective BCL2 targeting,^5^ identifying AML as a cancer with partial oncogenic dependence on BCL2,^12,15,16^ being able to deliver a BCL2 inhibitor systemically in combination with cytotoxic chemotherapy without causing indiscriminate toxicity,^25,27,38^ and culminating in the demonstration of improved OS in older, unfit AML despite negligible therapeutic progress using the same drugs as monotherapy.^31,98^ The difference that VEN has made in response rates and OS in elderly AML represents a landmark in improved care. It also represents a new starting point for redoubled efforts to improve cure rates in both old and young patients.

The full clinical potential of VEN and other BH3-mimetics in AML is only beginning to be explored. VEN and other BCL2 inhibitors that have recently entered clinical trials are being studied in fitter and younger patients in combination with a diverse array of standard and novel drugs. Some research questions with immediate importance include the following: (1) which agents should BCL2 inhibitors be combined with to improve outcomes for *TP53* mutated AML? (2) what strategies should be employed to prevent adaptive resistance in responders maintained on VEN regimens? (3) Is there a role for response-adapted, time-limited VEN regimens to reduce treatment burden without compromising benefit? and (4) If MCL1 inhibitors prove tolerable, how should they be combined with BCL2 inhibitors for maximum benefit in AML? Answers to these questions will greatly assist global efforts to build on the foundations laid over the last decade.

## ACKNOWLEDGMENTS

We acknowledge grants from the National Health and Medical Research Council of Australia, the Leukemia and Lymphoma Society (USA), Cancer Council of Victoria, Victorian Cancer Agency, Leukaemia Foundation of Australia, the Snowdome Foundation, the Medical Research Future Fund, Leukemia and Lymphoma Society (SCOR-Strasser) and the Metcalf Family Foundation.

## AUTHOR CONTRIBUTIONS

AW and AR did conceptualization; writing—original draft; and writing—review and editing.

## DISCLOSURES

AW and AR are employees of the Walter and Eliza Hall Institute, which receives milestone and royalty payments related to venetoclax; each receives a share of these royalties from the Institute. AR has received research funding to his institutions from Abbvie, Janssen, and Servier for investigator-initiated clinical trials or laboratory research. AW has served on advisory boards for Novartis, Janssen, Amgen, Roche, Pfizer, Abbvie, Servier, BMS, Gilead; receives research funding to the Institution from Novartis, Abbvie, Servier, BMS, Astra Zeneca, Amgen; serves on speaker bureaus for Abbvie, Novartis, and Celgene.

## SOURCES OF FUNDING

This work was supported by grants from the NHMRC of Australia, the Leukemia and Lymphoma Society (USA), Cancer Council of Victoria, Victorian Cancer Agency, Leukaemia Foundation of Australia, the Snowdome Foundation, the Medical Research Future Fund and the Metcalf Family Foundation.
